# Isolation and Characterization of Mosquito-Associated *Spiroplasma cantharicola* from *Aedes japonicus* Collected in Hokkaido, Japan

**DOI:** 10.3390/insects12121056

**Published:** 2021-11-25

**Authors:** Makoto Shimooka, Yoshimi Sakurai, Yasukazu Muramatsu, Leo Uchida

**Affiliations:** School of Veterinary Medicine, Rakuno Gakuen University, Ebetsu 069-8501, Hokkaido, Japan; s21761034@stu.rakuno.ac.jp (M.S.); s21561167@g.rakuno.ac.jp (Y.S.); y-mrmt@rakuno.ac.jp (Y.M.)

**Keywords:** *Aedes japonicus*, C6/36 cells, cytopathogenic effect, Japan, mosquito, *Spiroplasma cantharicola*

## Abstract

**Simple Summary:**

*Spiroplasma*, which comprises a group of the smallest known bacteria, is commonly found in insects and plants. Some species of *Spiroplasma* have shown pathogenicity against mosquitoes and are expected to be useful vector control tools. In this study, we isolated *S. cantharicola* from the mosquito *Aedes japonicus* collected in Hokkaido, northern Japan. Field surveillance implied a relatively low prevalence of the bacteria in the local mosquitoes. Phylogenetic analysis showed that our isolate is closer to a strain of *S. cantharicola* isolated from mosquitoes in previous studies. Our isolate grew well in the R2 broth medium and grew into a visible colony on R2 broth agar. Co-culturing with mosquito-derived C6/36 cells resulted in slightly more bacterial growth than culturing without cells, showing the strong cytopathogenic effect of the cells. To our knowledge, this is the third report on the isolation of *S. cantharicola* from mosquitoes.

**Abstract:**

Species of the genus *Spiroplasma* are common within arthropods and plants worldwide. Mosquito-associated *Spiroplasma* spp. have been reported to show pathogenicity toward mosquitoes, which serve as vectors of several infectious diseases that have detrimental effects on public health. Although *Spiroplasma* spp. are expected to have potential use as biological vector-control tools, characteristics such as their distribution, host species, and cytopathogenic effects (CPEs) are not well understood. In this study, we isolated a *Spiroplasma* sp. from a female *Aedes japonicus* collected in Hokkaido, northern Japan. Phylogenetic analysis based on the 16S rRNA gene sequence indicated our isolate was closely related to *S. cantharicola*. We screened 103 mosquito pools consisting of 3 genera and 9 species, but only detected *S. cantharicola* in the first isolation. In an in vitro assay, our isolate grew well at 28 °C, but no propagation was observed at 37 °C. Furthermore, the isolate showed strong CPE on a mosquito-derived cultured cell line (C6/36), and its propagation slightly increased when co-cultured with C6/36 cells. To our knowledge, this is the third report of the isolation of *S. cantharicola* from mosquitoes and the first case in Asia. Our findings provide epidemiological data on *S. cantharicola* distribution in the region.

## 1. Introduction

*Spiroplasmas* are some of the small and helical bacteria; they are self-replicating and are without a cell wall and flagellum [1]. They are categorized into the class *Mollicutes* (mostly comprising *Spiroplasma*, *Mycoplasma*, and *Acholeplasma* genera), and currently, almost 40 *Spiroplasma* species among 34 serological groups have been reported [1]. *Spiroplasmas* have mostly been found in arthropods, including mosquitoes and ticks, although plants and mammals are occasionally the hosts [2]. Generally, *Spiroplasmas* propagate in the midgut of arthropods, and in most cases, the bacteria are nonpathogenic to them. The pathogenicity of *Spiroplasma* seems to be related to the ability of their invasion beyond the epithelial cells of the midgut, i.e., some pathogenic species have been shown to expand their habitat to hemolymph, ovaries, fat bodies, hypodermis, or salivary glands [2].

Mosquitoes are one of the most important arthropods worldwide due to their vector competence for several pathogens, such as Dengue virus, Chikungunya virus, Rift Valley fever virus, and malaria parasites [3]. According to the American Mosquito Control Association, over one million people worldwide die annually from mosquito-borne diseases [4]. Furthermore, in most cases, effective vaccines and antiviral drugs are still under development, and antimalarial drug resistance is bringing further challenges [5,6]. Vector control efforts are also encountering difficulties due to the appearance of insecticide-resistant mosquitoes [7]. However, several mosquito symbionts, such as *Wolbachia* and *Spiroplasma*, are expected to be suitable for application as vector control tools [8,9,10,11,12]. Mosquito-associated *Spiroplasmas* are promising control tool candidates because of their pathogenicity to mosquitoes. The current list of mosquito-associated *Spiroplasmas* that have been characterized to species level is as follows: *S. culicola*, *S. taiwanense*, *S. sabaudiense*, *S. diminutum*, *S. cantharicola* [13], and *S. insolitum* [14]. A previously reported infectious in vivo experiment using four species of *Spiroplasma*, *S. culicola*, and *S. taiwanense* showed pathogenicity to *Aedes aegypti* mosquitoes, but all of them were nonpathogenic toward suckling mice and rats [15]. Additionally, the vertical transmission of *S. insolitum* in *Anopheles gambiae* was also proven recently [14], raising the possibility of the application of this *Spiroplasma* in the field such as an actual application of *Wolbachia* for reducing the Dengue incidence in a population [8,9,16].

In this study, we isolated a kind of arthropod-related *Spiroplasma*, *S. cantharicola*, from a female adult *Ae. japonicus* collected in Hokkaido, northern Japan. To our knowledge, this is the third report of *S. cantharicola* isolation from a mosquito [17,18]. Additionally, this report provides basic information on phylogenetics, as well as the growth kinetics when co-cultured with a mosquito-derived cell line.

## 2. Materials and Methods

### 2.1. Mosquito Sampling

From July 2016 to October 2020, adult mosquitoes were collected around Rakuno Gakuen University in Ebetsu City, Hokkaido Prefecture, Japan, using the human landing catch method and inst nets. The mosquitoes were initially collected for the isolation of arboviruses and were stored at −80 °C until further experiments.

In another experiment aimed at screening for *S. cantharicola*, 93 pools of previously and newly collected mosquitoes were used. From July to September 2016, adult *Ae. togoi* and *Ae. japonicus* were collected from around Otaru Aquarium in Otaru City, Hokkaido Prefecture. From June to October 2020, adult *Ae. japonicus*, *Ae. hokkaidensis*, *Culex pipiens* group, *Cx. orientalis*, and *An. sineroides* were collected from around Rakuno Gakuen University. From August to September 2020, adult *Ae. japonicus* and *Ae. galloisi* were collected in Nopporo Forest Park, Ebetsu City. All collection methods were the same as described above. As a control, 10 pools of insects from laboratory colonies of *Ae. albopictus* (Hatsudai linage) and *Ae. aegypti* (LIV-INB linage) were used for the study. The two species were kindly provided by Dr. Yuki Eshita of Hokkaido University Research Center for Zoonosis Control (Table S3).

Prior to the experiments, the mosquito species were identified based on their morphological characteristics according to Tanaka K. et al. [19]. On encountering difficulty with morphological identification, DNA barcoding based on the *COI* gene was used to support the species identification [20,21].

### 2.2. Isolation of Filtrable Microorganisms from Field-Collected Mosquitoes

*Ae. albopictus*-derived cultured cells (C6/36) were maintained at 28 °C under 5% CO_2_ in minimal essential medium (MEM) (Sigma-Aldrich, St. Louis, MO, USA) supplemented with 10% heat-inactivated fetal bovine serum (FBS), and an antibiotic cocktail of penicillin, streptomycin, and amphotericin B that was initially prepared for the purpose of isolating arboviruses [22].

For the isolation of arboviruses, mixtures of 1 mL of 2% FBS-MEM, field-collected mosquitoes, and a stainless bead were prepared in microtubes. The mosquitoes were homogenized with Micro Smash MS-100R (Tomy Digital Biology Co., Ltd., Tokyo, Japan) under the conditions of 4000 rpm for 20 s with cooling at 4 °C. The homogenates were centrifuged at 13,000 rpm for 1 min at 4 °C, and the supernatants were filtered with a 0.22-µm sterile syringe filter (Asone, Osaka, Japan) and syringe (Terumo, Tokyo, Japan). The supernatants were inoculated onto a C6/36 cell monolayer and incubated at 28 °C under 5% CO_2_ for 1 h. Afterward, the supernatant was removed, and 2% FBS-MEM was added to fill the well of the plate, followed by incubation in the same conditions. At 7 days post-infection (PI) the culture fluid was collected and inoculated onto a new C6/36 cell monolayer, and a blind passage was conducted a total of five times in duplicate. During the experiment, the cytopathogenic effects (CPEs) of the cells were observed daily, and the culture fluid of the CPE-positive samples was stored at −80 °C until further experiments.

### 2.3. DNA Extraction

DNA extraction from mosquito homogenate and culture fluid was conducted using the QIAamp DNA Mini Kit (Qiagen, Hilden, Germany) and/or NucleoSpin TriPrep (Macherey-Nagel, Düren, Germany) in accordance with the manufacturer’s protocols. Regarding the QIAamp DNA Mini Kit, the volume of elution buffer in the final extraction step was modified to between 50 µL and 200 µL, depending on the number of mosquitoes in the pool. The extracted DNA was stored at −30 °C until further experiments.

### 2.4. Polymerase Chain Reaction and Electrophoresis

**Figure 1:**
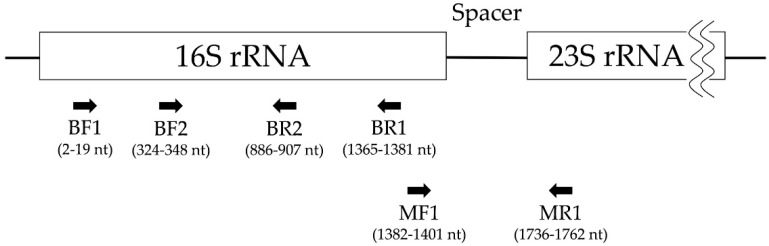
Map showing the location of primers on the 16S rRNA and 23S rRNA gene of *Spiroplasma*. Locations of the primers used in this study are shown by black arrows (Table S1). Nucleotide positions are based on *Spiroplasma* sp. (GenBank accession no. AJ245996.1).

To amplify the *Spiroplasma*-derived DNA, PCR was conducted using Takara Ex Taq (Takara Bio Inc., Shiga, Japan). The reaction mixture was modified to a total of 25 µL containing 0.125 µL of Takara Ex Taq (5 units/µL), 2.5 µL of 10 × Ex Taq Buffer, 2 µL of dNTP Mixture (2.5 mM), 0.5 µM each of forward and reverse primers, 5 µL of template DNA (concentration was not evaluated), and 12.875 µL of sterile distilled water. Two kinds of PCR were conducted for different purposes. The first primer set—BF1 and BR1, targeting universal bacterial 16S rRNA—was designed after referring to two previous studies, with some modification (Figure 1 and Table S1) [23,24]. The thermal cycling conditions were an initial denaturation at 94 °C for 2 min, followed by 30 cycles of denaturation at 98 °C for 10 s, annealing at 44.5 °C for 30 s, extension at 72 °C for 90 s, and a final extension at 72 °C for 10 min. The second primer set—MF1 and MR1, targeting a universal *Mycoplasma* spacer region between 16S rRNA and 23S rRNA—was designed after referring to the TaKaRa PCR *Mycoplasma* Detection Set (Takara Bio Inc., Shiga, Japan), with some modifications (Figure 1 and Table S1) [25]. The thermal cycling conditions were an initial denaturation at 94 °C for 2 min, followed by 40 cycles of denaturation at 94 °C for 30 s, annealing at 55 °C for 30 s, extension at 72 °C for 60 s, and a final extension at 72 °C for 10 min. The specificity of the primer set MF1 and MR1 was confirmed using positive control DNA extracted from our isolate before it was used for screening.

The amplicons were loaded onto 1.5% agarose gel containing 0.01% of ethidium bromide and electrophoresed at 7.4 V/cm for 30–40 min in 1x TBE buffer (50 mM Tris-HCl, 48.5 mM boric acid, 2 mM EDTA, pH 8.0).

### 2.5. Sequencing and Genetic Analysis

The amplicons were purified using the Fast Gene PCR Extraction Kit (Nippon Genetics Co., Ltd., Kanagawa, Japan), and the nucleotide sequences were determined by the FASMAC sequence service (Fasmac Co., Ltd., Kanagawa, Japan) using the above PCR primer sets and two additional sequencing primers, BF2 and BR2, specific to sequences located between the BF1 and BR1 annealing sites (Figure 1 and Table S1) [26,27]. We searched for sequences with similarity using the basic local alignment search tool (BLAST) (https://blast.ncbi.nlm.nih.gov/Blast.cgi (accessed on 15 June 2021)). Contig sequences were generated by the sequence analysis software GeneStudio Professional v2.2.0.0. To conduct phylogenetic analysis, reference sequence data were collected from GenBank (Table S2), and multiple alignments were constructed by ClustalW v2.1. Phylogenetic trees based on the neighbor-joining method were created with 1000 bootstrap replicates, and the tree was visualized with the tree figure drawing tool FigTree v1.4.4. In genetic analysis aiming to compare sequence similarities among our isolate and reference sequences, the pairwise alignment tool EMBOSS Needle (https://www.ebi.ac.uk/Tools/psa/emboss_needle/ (accessed on 8 June 2021)) was used to obtain the global alignment.

### 2.6. Culturing of S. cantharicola

R2 broth medium, which is a simplified media for culturing *Spiroplasma* bacteria, was prepared in accordance with a previous study [28]. To make agar plates, 1.5% agarose was added to the R2 broth medium, and the plate was used for the titration of *S. cantharicola*.

First, the cell culture fluid that showed CPE on C6/36 cells was inoculated onto a PPLO agar plate (Nikkenseibutsu K.K., Kyoto, Japan), and a single colony was picked after incubating the plate at 28 °C. The isolated single colony was inoculated into 7 mL of R2 broth medium [28], which was incubated at 28 °C under 5% CO_2_ for propagation. When the color of the medium turned orange to yellow (approximately 5 days PI), the propagated bacteria were collected, placed into a medium in microtubes, and stored at −80 °C. For the titration of the bacteria, 10-fold serially diluted medium was inoculated onto R2 agar plates, and the plates were incubated at 28 °C under 5% CO_2_. Three to 4 weeks later, visible colonies were counted, from which the number of bacteria (colony-forming units, CFU/mL) was calculated. In the growth curve assay, 1.0 × 10^3^ CFU/mL of bacteria was prepared in R2 broth at the starting point, and the plates were incubated at 28 °C or 37 °C under 5% CO_2_. The culture fluid was harvested every 24 h for up to 7 days PI, and the bacteria titer was determined using the above methods.

In another experiment, our isolate was co-cultured with C6/36 cells to evaluate the effects of the cells on the isolate. Cell monolayers were prepared in 96-well plates, and the bacteria were spiked into 2% FBS-MEM at a multiplicity of infection of 0.002. The plates were incubated at 28 °C under 5% CO_2_, and the bacteria titer was determined following the above method.

### 2.7. Statistical Analyses

To evaluate the significant differences of bacteria titers, statistical analyses were performed by Microsoft Excel 2016 software. F-test was used to confirm whether the variance among the samples derived from different replicates was equal. According to the F-test results, an unpaired two-tailed Student’s *t*-test with the condition of equal variances was conducted to compare the bacteria titers. P value below 0.05 was considered statistically significant.

## 3. Results

### 3.1. Isolation and Identification of Filtrable Microorganism from a Field-Collected Mosquito

**Figure 2:**
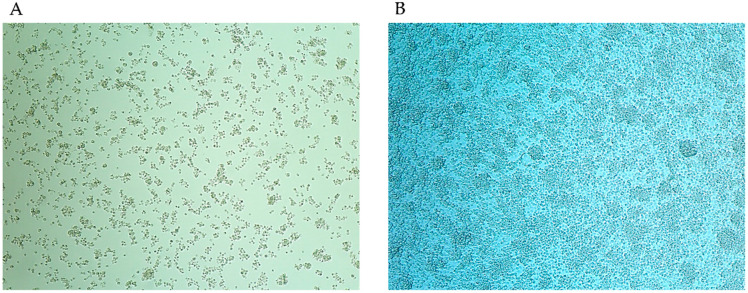
Cytopathogenic effect of our isolate on C6/36 cells. Mosquito-derived homogenate caused strong CPE on C6/36 cells (**A**). The mock control does not show any CPE (**B**). The experiment was conducted in duplicate, and the cell culture fluid was blindly passed five times in total.

**Figure 3:**
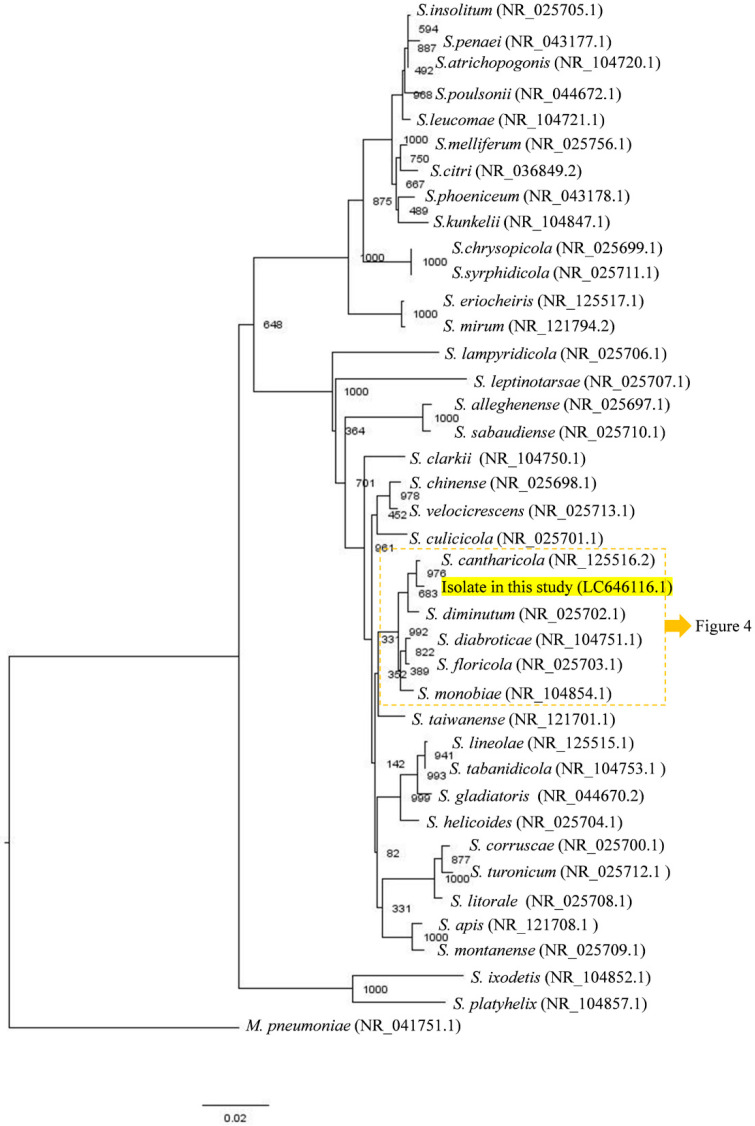
Phylogenetic tree showing the relationship between the *Spiroplasma* sp. isolated in this study and other *Spiroplasmas*. Approximately 1250 bp of the 16S rRNA nucleotide sequences of the genus *Spiroplasma* were aligned by ClustalW with 1000 bootstrap replicates, and the tree was visualized by FigTree. All *Spiroplasma* species that have been determined to the species name were included in the tree. The available sequences were selected from NCBI Reference Sequence Database, and *Mycoplasma pneumoniae* was added as an outgroup. The numbers in the nodes indicate bootstrap values. Candidatus *Spiroplasma holothuricol* was excluded from the analysis.

**Figure 4:**
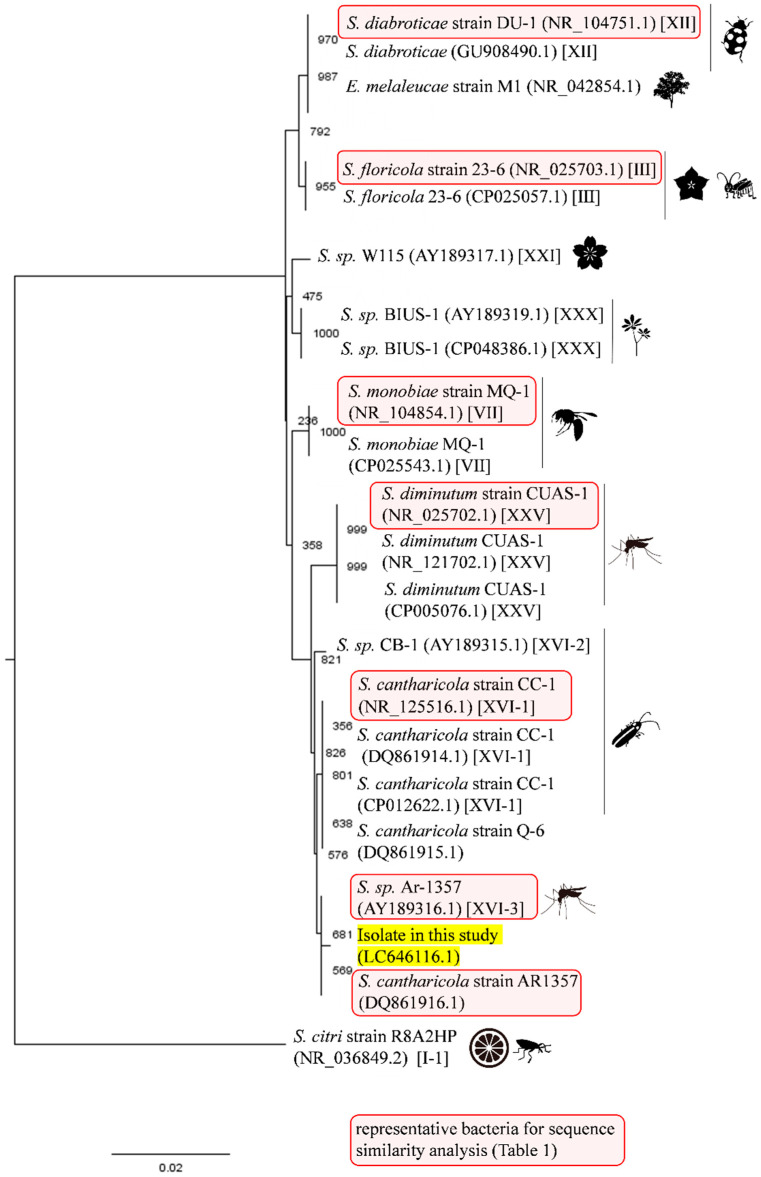
Phylogenetic tree of the *Spiroplasma* sp. isolated in this study and the top 20 strains from a BLAST search. Approximately 1283 bp of 16S rRNA nucleotide sequences from the top 20 most similar *Spiroplasma* strains were aligned by ClustalW with 1000 bootstrap replicates. The image was visualized by FigTree. The Roman numerals in brackets show serogroups reported in a previous study (Table S2). Illustrations in addition to the labels indicate the hosts of the bacteria (Table S2) [2]. *S. citri* was added as an outgroup. The numbers in the nodes indicate bootstrap values.

During the attempt to isolate arboviruses, one pool consisted of a female *Ae. japonicus* showed CPE on C6/36 cells (Figure 2). Although we could not identify any viral RNA (pan-Flavivirus or pan-Phenuiviridae) in the supernatant, we suspected the presence of filtrable microorganisms that could pass through a 0.22 µm filter; thus, we initially suspected Mycoplasma. To identify the unknown microorganisms, conventional PCR based on the 16S rRNA sequence was conducted. As a result, a size-specific amplicon was obtained, and phylogenetic analysis revealed our isolate to be closely related to *S. cantharicola* (Figure 3). Furthermore, detailed phylogenetic analysis showed that our isolate was closer to *S. cantharicola* strain AR1357 (DQ861916.1) and *Spiroplasma* sp. Ar-1357 (AY189316.1), which were isolated from Aedes mosquitoes, then to S. cantharicola strain CC-1 (NR125516.1) isolated from Cantharis carolinus (Figure 4). Global alignment analysis by EMBOSS Needle showed 99.8% sequence similarity between our isolate and both the *S. cantharicola* strain AR1357 (DQ861916.1) and *Spiroplasma* sp. Ar-1357 (AY189316.1) (Table 1). However, the registered sequences of *S. cantharicola* strain AR1357 (DQ861916.1) and *Spiroplasma* sp. Ar-1357 (AY189316.1) have 99.8% similarity. Taken together, these findings show our isolate belongs to *S. cantharicola* and is closely related to mosquito-derived strains. The determined sequence of our isolate was deposited in the GenBank with accession no. LC646116.1 and LC646117.1.

### 3.2. Prevalence of S. cantharicola in the Field-Collected Mosquitoes and Laboratory Colonies

To clarify the field prevalence of *S. cantharicola* in northern Japan, an epidemiological survey based on PCR was conducted using MF1 and MR1 primer sets. Prior to the assay, the specificity of the PCR was confirmed using our isolate and cell culture fluid derived from uninfected C6/36 cells. A total of 93 pools consisting of 614 field-collected mosquitoes (3 genera, 9 species) were evaluated by PCR, but no pools were positive (Table S3). Similarly, *S. cantharicola* DNA was not detected from the two laboratory colonies of Ae. albopictus and Ae. aegypti (Table S3).

### 3.3. Growth Kinetics of S. cantharicola

**Figure 5:**
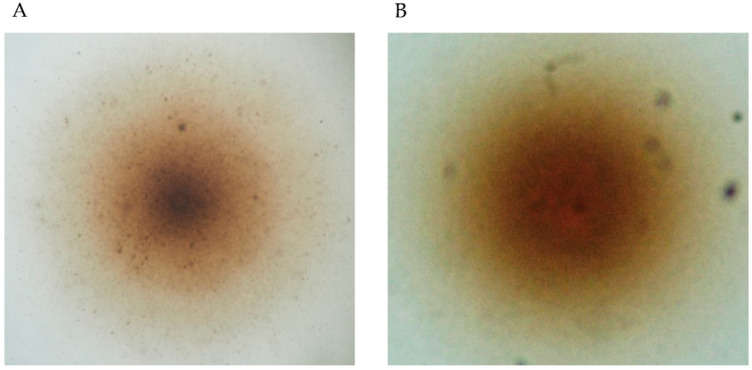
Colony on the PPLO and R2 agar plates. The cell culture supernatant that showed CPE on C6/36 was inoculated onto a PPLO agar plate and incubated at 28 °C under 5% CO_2_. At 15 days PI, an image of a single colony was captured with a digital camera (**A**). The single colony on the PPLO agar plate was picked, and plated onto R2 agar, and incubated in the same manner as the PPLO agar plate. An image of a single colony on R2 agar plate was captured at 19 days PI (**B**).

Prior to the evaluation of growth kinetics, the colony formation ability of our isolate was confirmed with PPLO agar plates and R2 agar plates (Figure 5A,B). In both plates, our isolate grew into visible colonies by 3 to 5 days PI. The speed of colonization was faster on PPLO agar than on R2 agar, but the colony size was almost equal between the two plates.

**Figure 6:**
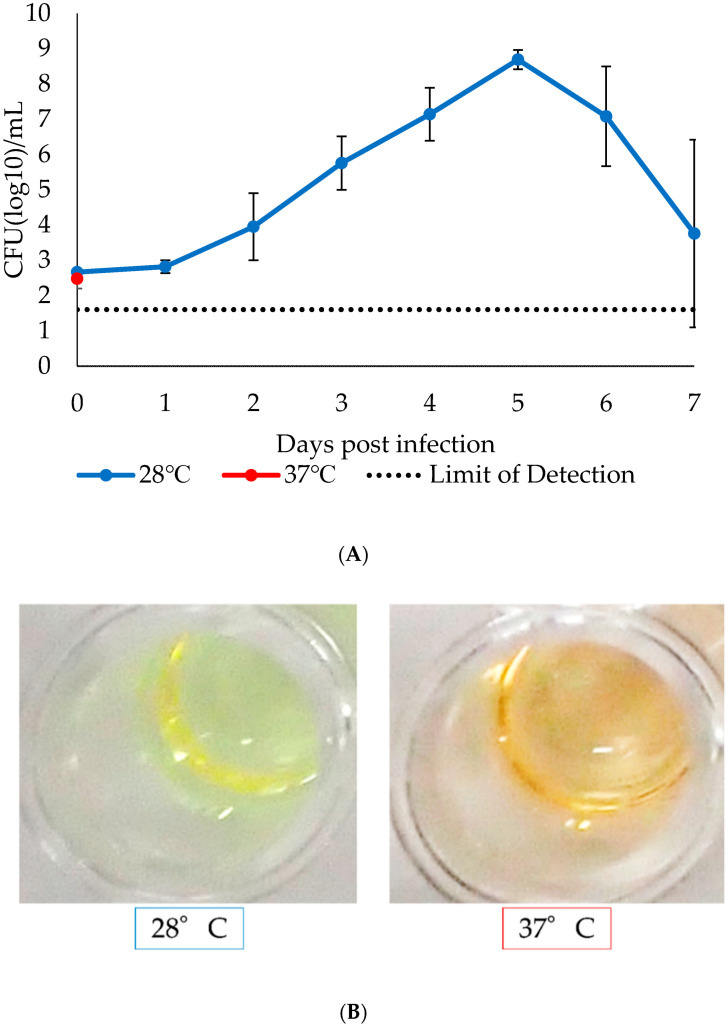
Growth curve of our isolate in R2 broth medium and color change of the medium. Bacteria at 10^3^ CFU/mL in R2 broth medium were placed into 96-well plates and incubated at 28 °C or 37 °C under 5% CO_2_. The culture fluid was collected daily, and the bacteria titer was determined using R2 agar (**A**). The experiments were conducted twice independently, and the average titer was calculated. Error bars in the graph indicate standard error, and the dotted lines show detection limit of the assay. The color change of R2 broth medium at 7 days PI is shown in (**B**).

To evaluate the effect of temperature on the growth kinetics, we conducted growth curve analysis in the R2 broth medium at two temperatures, 28 °C and 37 °C, to replicate arthropod and mammal environmental conditions, respectively. Although the bacteria propagated efficiently at 28 °C, those grown at 37 °C could not be detected; that is, no colonies were observed from the culture fluid collected 1-day PI at 37 °C. The number of bacteria increased exponentially at 28 °C by 4 days PI, but afterward, the titer slowly decreased (Figure 6A). The color difference of the R2 broth medium was also significant between the culture at 28 °C and that at 37 °C, as the former resulted in a change from orange to yellow at around the growth plateau, but the latter stayed orange throughout the culture (Figure 6B).

**Figure 7:**
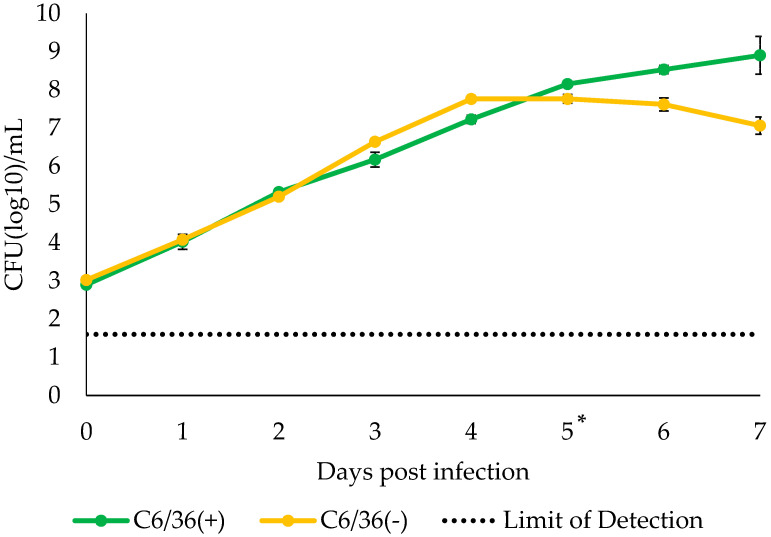
Growth curve of our isolate co-cultured with C6/36 cells. Bacteria at 10^3^ CFU/mL were spiked into 2% FBS-MEM and incubated at 28 °C under 5% CO_2_ with or without C6/36 cells. The supernatant was collected daily, and the bacteria titer was determined using R2 agar. The experiments were conducted twice independently and the average titer was calculated. Error bars in the graph indicate standard error, and the dotted lines show limit of detection in the assay. Asterisk in the figure indicates the point that C6/36 cells started to show CPE.

Finally, the effect of C6/36 cells on bacteria growth kinetics was evaluated. The titer of the bacteria in the supernatant showed different growth trends under the presence or absence of the cells. Those cultured without C6/36 cells showed higher titers from 3 to 4 days PI but started to decrease after 5 days PI. In contrast, those cultured with C6/36 showed higher titers between 5 and 7 days PI, and the high levels persisted longer (Figure 7). However, contrary to our expectation, the *t*-test analysis of the bacteria titers did not show any significant differences. Moreover, C6/36 cells began to show CPE identical to those in Figure 2 after 5 days PI.

## 4. Discussion

In this study, we isolated a filtrable microorganism from *Ae. japonicus* collected in Hokkaido, northern Japan. Sequence analysis based on the 16S rRNA gene showed our isolate had quite high similarities to *S. cantharicola* (Table 1). Among *S. cantharicola* strains, our isolate was closer to strain Ar-1357 (*Aedes* mosquito-derived strain) at 99.8% sequence similarity, than strain CC-1 (*C. carolinus*-derived strain) (Figure 4, Table 1). Strain Ar-1357 was isolated from *Ae. cantans*/*vexans* in Savoy, France, from 1983 to 1985 [17,29], and strain CC-1 was isolated from *C. carolinus* in Maryland, USA, in 1982 [18]. To our knowledge, this is the third report of the isolation of *S. cantharicola* from mosquitoes in Asia. It is intriguing that there have not been more reports of the isolation of *S. cantharicola* because many scientists have made efforts to find arboviruses using mosquito-derived cells. One hypothesis to answer this question could be a lack of interest in the bacteria, and any strains may have been treated as simple contamination during virus isolation. *Ae. japonicus* originating from East Asia has been detected in the USA since 1998 and France since 2000, and it has been declared as an invasive species in these countries [30,31]. Similar to other mosquito-borne viruses, such as the Zika virus and West Nile virus, it is possible that *Spiroplasma* was introduced into new areas that have not kept isolation records.

In the growth curve assay, our isolate grew exponentially at 28 °C in R2 broth medium (one of the simplest broth media for *Spiroplasmas*), similar to *S. helicoides* and *S. litorale* in the same medium at 30 °C [28], but our isolate did not grow at 37 °C (Figure 6). Abalain-Colloc M.L. et al. also reported that *Spiroplasma* sp. strain Ar-1357 grew well at 10−32 °C but not at 37 °C [29]. In this study, we used the temperatures 28 °C and 37 °C, which are typically used for arthropod- and mammalian-derived cell culture, respectively. In previously reported in vivo infection experiments, mice and rats were challenged with four mosquito-associated *Spiroplasmas* (*S. culicola*, *S. taiwanense*, *S. subaudiense*, and *S. diminutum*) [15]; the bacteria did not cause any symptoms or death, and although *S. culicicola* persisted in the brain for up to 14 days PI, three *Spiroplasma* sp. could not be re-isolated [15]. In fact, the optimum growth temperature of *S. culicola* in vitro was 37 °C, but that of the other three bacteria was between 30 °C and 32 °C, showing the correlation between the in vivo and in vitro assays [32]. Collectively, the evidence suggests that our isolate would neither propagate in mammals nor persist in mammalian tissue, even when directly inoculated.

The effect of C6/36 cells on our isolate was not fully unveiled, but our data showed the possibility that they may have had a positive effect on the bacteria’s growth. According to the growth curve analysis using the supernatant from the culture with or without C6/36 cells, the bacteria titer without C6/36 cells was higher than that of the culture with the cells before the CPE of the C6/36 cells appeared (5 days PI), but afterward, the trend was reversed, and the higher titers of those cultured with C6/36 cells persisted for 7 days PI (Figure 7). To date, the infectivity of *S. cantharicola* to C6/36 cells—the internalization of the bacteria into the cells—is still unclear. Duret S. et al. reported transmission electron microscopy-captured images of the internalization of *S. citri*, a species that infects both arthropods and plants, into leafhopper (*Neoaliturus haematoceps*)-derived Ciha-1 cells [33]. The internalization occurs through receptor-mediated endocytosis [34,35]. In another study, Humphery-Smith et al. found that *S. sabaudiense* induced the lysis of C6/36 cells after several passages with the cells, suggesting that the CPE was a cell-type-specific phenomenon, not the results of simple contamination, as seen with other bacteria [36]. Similar data on the modulation of pathogenicity have been reported by Steiner et al. using *S. citri* [37]. One possible scenario to explain the enhanced growth of our isolate when it was co-cultured with C6/36 cells is that the bacteria used cellular factors as nutrients after internalization into the cells. As shown in Figure 6A and a previous study [18], *S. cantharicola* does not require any cellular components for its growth, but if intracellular factors are available as nutrients, replication may be increased. In our preliminary experiments, our isolate did not show CPE on Vero cells, but it was considered to be the result of the bacteria’s inability to grow at 37 °C (data not shown). Multidimensional analysis based on culture with several types of arthropod cell lines is needed to evaluate the effect of cellular components on the bacteria.

Recently, several challenges of the use of mosquito symbionts, such as *Wolbachia* and *Spiroplasma*, as vector control tools have been attempted [8,9,10,12,38]. These bacteria have been used to kill the arthropod, reduce its reproductive ability, and diminish the vector competence [12]. As outlined above, some species of *Spiroplasma* have pathogenicity to mosquitoes [11,15], but their effect on the vector competence of arboviruses is completely unknown. A promising aspect of using vector control pathogens is the possibility of vertical transmission. Chepkemoi et al. reported that *S. insolitum* was maintained in *An. gambiae* was vertically transmitted over at least two generations [14]. To date, basic information, such as the effect on vector competence and vertical transmission of *S. cantharicola*, is sparse, while similar information for other mosquito-associated *Spiroplasmas* such as *S. culicicola*, *S. taiwanense*, *S. diminutum*, and *S. sabaudiense* is only partly available [11]. In addition to evaluations in arthropods, the influence of the pathogens on plants and mammals must be evaluated, as positive sera against *Spiroplasma* sp. have been reported in cows [2,17].

In conclusion, this study provides basic information on the distribution of mosquito-associated *S. cantharicola* in northern Japan and the basic bacteria characteristics in vitro. Our data contribute to the understanding of this bacteria and will aid its application in further studies.

## Figures and Tables

**Table 1:** Species similarities based on 16S rRNA sequences from the *Spiroplasma* sp. isolated in this study and other representative bacteria.

	Isolate in This Study (LC646116.1)	*S. cantharicola* Strain AR1357 (DQ861916.1)	*Spiroplasma* sp. Ar-1357 (AY189316.1)	*S. cantharicola* Strain CC-1 (NR_125516.1)	*S. diminutum* Strain CUAS-1 (NR_025702.1)	*S. monobiae* Strain MQ-1 (NR_104854.1)	*S. floricola* Strain 23-6 (NR_025703.1)
*S. cantharicola* strain AR1357 (DQ861916.1)	99.8% (1281/1283)						
*Spiroplasma* sp. Ar-1357 (AY189316.1)	99.8% (1281/1283)	99.8% (1502/1505)					
*S. cantharicola* strain CC-1 (NR_125516.1)	99.7% (1279/1283)	99.9% (1503/1505)	99.7% (1500/1504)				
*S. diminutum* strain CUAS-1 (NR_025702.1)	99.2% (1273/1283)	98.9% (1351/1366)	99.4% (1507/1516)	99.3% (1493/1504)			
*S. monobiae* strain MQ-1 (NR_104854.1)	99.1% (1272/1283)	99.3% (1456/1467)	99.3% (1457/1467)	99.2% (1455/1467)	98.9% (1451/1467)		
*S. floricola* strain 23-6 (NR_025703.1)	98.8% (1268/1283)	98.9% (1351/1366)	99.0% (1364/1378)	98.9% (1351/1366)	99.0% (1364/1378)	99.4% (1355/1363)	
*S. diabroticae* strain DU-1 (NR_104751.1)	98.8% (1267/1283)	98.9% (1487/1504)	99.0% (1493/1508)	98.9% (1487/1504)	99.2% (1496/1508)	99.3% (1457/1467)	99.7% (1366/1370)

## Data Availability

The reference sequence data used in this study are openly available at GenBank (https://www.ncbi.nlm.nih.gov/genbank/ (accessed on 12 September 2021)). The determined sequence of our isolate was deposited in GenBank with accession no. LC646116.1 and LC646117.1.

## Supplementary Materials

The following are available online at https://www.mdpi.com/article/10.3390/insects12121056/s1: Table S1: Primers used in this study, Table S2: Detailed information on bacteria in the phylogenetic tree, Table S3: Detailed information of samples.

## References

[1] Cisak E., Wójcik-Fatla A., Zając V., Sawczyn A., Sroka J., Dutkiewicz J. (2015). Spiroplasma-an emerging arthropod-borne pathogen?. Ann. Agric. Environ. Med..

[2] Regassa L.B., Gasparich G.E. (2006). Spiroplasmas: Evolutionary relationships and biodiversity. Front. Biosci..

[3] Lee H., Halverson S., Ezinwa N. (2018). Mosquito-borne diseases. Prim. Care.

[4] AMCA Mosquito-Borne Diseases. https://www.mosquito.org/page/diseases.

[5] Gould E.A., Solomon T. (2008). Pathogenic flaviviruses. Lancet.

[6] White N.J. (2004). Antimalarial drug resistance. J. Clin. Investig..

[7] Ranson H., N’Guessan R., Lines J., Moiroux N., Nkuni Z., Corbel V. (2011). Pyrethroid resistance in african anopheline mosquitoes: What are the implications for malaria control?. Trends Parasitol..

[8] Hoffmann A.A., Montgomery B.L., Popovici J., Iturbe-Ormaetxe I., Johnson P.H., Muzzi F., Greenfield M., Durkan M., Leong Y.S., Dong Y. (2011). Successful establishment of wolbachia in aedes populations to suppress dengue transmission. Nature.

[9] Moreira L.A., Iturbe-Ormaetxe I., Jeffery J.A., Lu G., Pyke A.T., Hedges L.M., Rocha B.C., Hall-Mendelin S., Day A., Riegler M. (2009). A wolbachia symbiont in aedes aegypti limits infection with dengue, chikungunya, and plasmodium. Cell.

[10] Humphery-Smith I., Grulet O., Chastel C. (1991). Pathogenicity of spiroplasma taiwanense for larval aedes aegypti mosquitoes. Med. Vet. Entomol..

[11] Humphery-Smith I., Grulet O., Le Goff F., Chastel C. (1991). Spiroplasma (mollicutes: Spiroplasmataceae) pathogenic for aedes aegypti and anopheles stephensi (diptera: Culicidae). J. Med. Entomol..

[12] Jupatanakul N., Sim S., Dimopoulos G. (2014). The insect microbiome modulates vector competence for arboviruses. Viruses.

[13] Chang T.H., Lo W.S., Ku C., Chen L.L., Kuo C.H. (2014). Molecular evolution of the substrate utilization strategies and putative virulence factors in mosquito-associated spiroplasma species. Genome Biol. Evol..

[14] Chepkemoi S.T., Mararo E., Butungi H., Paredes J., Masiga D., Sinkins S.P., Herren J.K. (2017). Identification of spiroplasmainsolitum symbionts in anopheles gambiae. Wellcome Open Res..

[15] Vazeille-Falcoz M., Perchec-Merien A.M., Rodhain F. (1994). Experimental infection of aedes aegypti mosquitoes, suckling mice, and rats with four mosquito spiroplasmas. J. Invertebr. Pathol..

[16] Utarini A., Indriani C., Ahmad R.A., Tantowijoyo W., Arguni E., Ansari M.R., Supriyati E., Wardana D.S., Meitika Y., Ernesia I. (2021). Efficacy of wolbachia-infected mosquito deployments for the control of dengue. N. Engl. J. Med..

[17] Chastel C., Devau B., Le Goff F., Simitzis-Le Flohic A.M., Gruffaz R., Kerdraon G., Gilot B. (1987). Mosquito spiroplasmas from france and their ecology. Isr. J. Med. Sci..

[18] Whitcomb R.F., Chastel C., Abalain-Colloc M., Stevens C., Tully J.G., Rose D.L., Carle P., Bové J.M., Henegar R.B., Hackett K.J. (1993). Spiroplasma cantharicola sp. Nov., from cantharid beetles (coleoptera: Cantharidae). Int. J. Syst. Evol. Microbiol..

[19] Tanaka K., Mizusawa K., Saugstad E.S. (1979). A revision of the adult and larval mosquitoes of japan (including the ryukyu archipelago and the ogasawara islands) and korea (diptera: Culicidae). Contrib. Am. Entomol. Inst..

[20] Folmer O., Black M., Hoeh W., Lutz R., Vrijenhoek R. (1994). DNA primers for amplification of mitochondrial cytochrome c oxidase subunit i from diverse metazoan invertebrates. Mol. Mar. Biol. Biotechnol..

[21] Uchida L., Shibuya M., Morales-Vargas R.E., Hagiwara K., Muramatsu Y. (2021). Zika virus potential vectors among aedes mosquitoes from hokkaido, northern japan: Implications for potential emergence of zika disease. Pathogens.

[22] Igarashi A. (1978). Isolation of a singh’s aedes albopictus cell clone sensitive to dengue and chikungunya viruses. J. Gen. Virol..

[23] Fredriksson N.J., Hermansson M., Wilen B.M. (2013). The choice of pcr primers has great impact on assessments of bacterial community diversity and dynamics in a wastewater treatment plant. PLoS ONE.

[24] Beckers B., Op De Beeck M., Thijs S., Truyens S., Weyens N., Boerjan W., Vangronsveld J. (2016). Performance of 16s rdna primer pairs in the study of rhizosphere and endosphere bacterial microbiomes in metabarcoding studies. Front. Microbiol..

[25] Uemori T., Asada K., Kato I., Harasawa R. (1992). Amplification of the 16s-23s spacer region in rrna operons of mycoplasmas by the polymerase chain reaction. Syst. Appl. Microbiol..

[26] Hatamoto M., Imachi H., Ohashi A., Harada H. (2007). Identification and cultivation of anaerobic, syntrophic long-chain fatty acid-degrading microbes from mesophilic and thermophilic methanogenic sludges. Appl. Environ. Microbiol.

[27] Lazarevic V., Gaia N., Girard M., Schrenzel J. (2016). Decontamination of 16s rrna gene amplicon sequence datasets based on bacterial load assessment by qpcr. BMC Microbiol..

[28] Moulder R.W., French F.E., Chang C.J. (2002). Simplified media for spiroplasmas associated with tabanid flies. Can. J. Microbiol..

[29] Abalain-Colloc M.L., Williamson D.L., Carle P., Abalain J.H., Bonnet F., Tully J.G., Konai M., Whitcomb R.F., Bové J.M., Chastel C. (1993). Division of group xvi spiroplasmas into subgroups. Int. J. Syst. Evol. Microbiol..

[30] Koban M.B., Kampen H., Scheuch D.E., Frueh L., Kuhlisch C., Janssen N., Steidle J.L.M., Schaub G.A., Werner D. (2019). The asian bush mosquito aedes japonicus japonicus (diptera: Culicidae) in europe, 17 years after its first detection, with a focus on monitoring methods. Parasit. Vectors.

[31] Peach D.A.H., Almond M., Pol J.C. (2019). Modeled distributions of aedes japonicus japonicus and aedes togoi (diptera: Culicidae) in the united states, canada, and northern latin america. J. Vector Ecol..

[32] Konai M., Clark E.A., Camp M., Koeh A.L., Whitcomb R.F. (1996). Temperature ranges, growth optima, and growth rates of spiroplasma (spiroplasmataceae, class mollicutes) species. Curr. Microbiol..

[33] Duret S., Batailler B., Dubrana M.P., Saillard C., Renaudin J., Béven L., Arricau-Bouvery N. (2014). Invasion of insect cells by spiroplasma citri involves spiralin relocalization and lectin/glycoconjugate-type interactions. Cell Microbiol..

[34] Fletcher J., Wayadande A., Melcher U., Ye F. (1998). The phytopathogenic mollicute-insect vector interface: A closer look. Phytopathology.

[35] Kwon M.O., Wayadande A.C., Fletcher J. (1999). Spiroplasma citri movement into the intestines and salivary glands of its leafhopper vector, circulifer tenellus. Phytopathology.

[36] Humphery-Smith I., Grulet O., Le Lay G., Chastel C. (1988). Pathogenicity of spiroplasma sabaudiense (mollicute) for the cells (c6/36) of aedes albopictus (insecta: Diptera) in vitro. Bull. Soc. Pathol. Exot. Fil..

[37] Steiner T., McGarrity G.J., Phillips D.M. (1982). Cultivation and partial characterization of spiroplasmas in cell cultures. Infect. Immun..

[38] Abalain-Colloc M.L., Rosen L., Tully J.G., Bovë J.M., Chastel C., Williamson D.L. (1988). Spiroplasma taiwanense sp.Nov. From culex tritaeniorhynchus mosquitoes collected in taiwan. Int. J. Syst. Evol. Microbiol..

