# Supplementary material for: Isolation and Characterization of Mosquito-Associated Spiroplasma cantharicola from Aedes japonicus Collected in Hokkaido, Japan
Source: Insects. 2021 Nov 25;12(12):1056. doi: 10.3390/insects12121056 (PMC8703607; doi:10.3390/insects12121056)
Supplement: Supplementary file 1 [file insects-12-01056-s001.zip › insects-1441669-supplymentary materials.pdf]

Supplementary information

Table S1. Primers used in this study

Purpose	Primer name	Sequence (5'-3')*	Region
Identification of *Spiroplasma* isolated in this study	BF1	AGAGTTTGATCMTGGCTC	16S rRNA
	BF2	ACTCCTACGGGAGGCAGCAGTRGGG	
	BR2	CCCCGTCAATTCMTTTGAGTTT	
	BR1	GACGGGCGGTGWGTRCA	
Detection of *Spiroplasma* from mosquitoes for prevalence	MF1	ACACCATGGGAGCTGGTAAT	16S-23S Spacer
	MR1	CTTCWTCGACTTYCAGACCCAAGGCAT	

*M: A or C, R: A or G, W: A or T, Y: C or T

Table S2. Detailed information on bacteria in the phylogenetic tree

Species, strain, (accession no.)	Serotype[a]	Host	
S. diabroticae strain DU-1 (NR_104751.1)	[XII]		*Diabrotica undecimpunctata* beetles[a]
S. diabroticae (GU908490.1)	[XII]		
E. melaleucae strain M1 (NR_042854.1)		*Melaleuca quinquenervia*[b]	
S. floricola strain 23-6 (NR_025703.1)	[III]		Insects, flower[a]
S. floricola 23-6 (CP025057.1)	[III]	*Liriodendron tulipifera*[b]	
Spiroplasma sp. W115 (AY189317.1)	[XXI]		*Prunus* sp. flowers[a]
Spiroplasma sp. BIUS-1 (AY189319.1)	[XXX]		Flower surface[a]
Spiroplasma sp. BIUS-1 (CP048386.1)	[XXX]	*Bidens* sp.[b]	
S. diminutum CUAS-1 (NR_121702.1)	[XXV]		*Culex* mosquito[a]
S. diminutum strain CUAS-1 (NR_025702.1)	[XXV]		
S. diminutum CUAS-1 (CP005076.1)	[XXV]		
Spiroplasma sp. CB-1 (AY189315.1)	[XVI-2]		Cantharid beetle[a]
S. cantharicola strain CC-1	[XVI-1]	Cantharis[b]	

(CP012622.1)			
S. cantharicola strain Q-6 (DQ861915.1)			
S. cantharicola strain CC-1 *(NR_125516.1)*	[XVI-1]		Cantharid beetle[a]
S. cantharicola strain CC-1 (DQ861914.1)	[XVI-1]		
Spiroplasma sp. Ar-1357 *(AY189316.1)*	[XVI-3]		Mosquito[a]
*S. cantharicol*a strain AR1357 (DQ861916.1)			
S. monobiae MQ-1 (CP025543.1)	[VII]	*Monobia quadridens*[b]	*Monobia* wasps[a]
S. monobiae strain MQ-1 (NR_104854.1)	[VII]		
S. citri strain R8A2HP (NR_036849.2)	[I-1]		Dicots, leafhoppers[a]

[a]Information on serotype and host was retrieved from Regassa L.B. *et al* [2].

[b]Information on host was retrieved from Genbank site because it was not available in the reference [2].

Table S3. Detailed information of samples

Mosquito Pool ID	Species	Sex	Number of Mosquitoes	Collection site	Collection date	PCR	BLAST search result
1	*Aedes togoi*	Male	1	Otaru aquarium	11th Aug 2016	-	
2	*Aedes togoi*	Male	3	Otaru aquarium	11th Aug 2016	-	
3	*Aedes togoi*	Male	5	Otaru aquarium	25th Aug 2016	-	
4	*Aedes togoi*	Male	20	Otaru aquarium	25th Aug 2016	-	
5	*Aedes togoi*	Male	8	Otaru aquarium	25th Aug 2016	-	
6	*Aedes togoi*	Male	1	Otaru aquarium	12th Sep 2016	-	
7	*Aedes togoi*	Male	9	Otaru aquarium	12th Sep 2016	-	
8	*Aedes togoi*	Male	20	Otaru aquarium	12th Sep 2016	-	
9	*Aedes togoi*	Male	2	Otaru aquarium	12th Sep 2016	-	
10	*Aedes togoi*	Male	8	Otaru aquarium	27th Sep 2016	-	
11	*Aedes togoi*	Female	5	Otaru aquarium	20th Jul 2016	-	
12	*Aedes togoi*	Female	1	Otaru aquarium	20th Jul 2016	-	
13	*Aedes togoi*	Female	4	Otaru aquarium	20th Jul 2016	-	
14	*Aedes togoi*	Female	6	Otaru aquarium	11th Aug 2016	+	nonspecific reaction
15	*Aedes togoi*	Female	1	Otaru aquarium	25th Aug 2016	-	
16	*Aedes togoi*	Female	17	Otaru aquarium	25th Aug 2016	-	
17	*Aedes togoi*	Female	2	Otaru aquarium	12th Sep 2016	+	nonspecific reaction
18	*Aedes togoi*	Female	1	Otaru aquarium	12th Sep 2016	-	
19	*Aedes togoi*	Female	3	Otaru aquarium	12th Sep 2016	-	

20	*Aedes togoi*	Female	7	Otaru aquarium	12th Sep 2016	-	
21	*Aedes togoi*	Female	14	Otaru aquarium	12th Sep 2016	-	
22	*Aedes togoi*	Female	1	Otaru aquarium	27th Sep 2016	-	
23	*Aedes togoi*	Female	1	Otaru aquarium	27th Sep 2016	-	
24	*Aedes togoi*	Female	15	Otaru aquarium	27th Sep 2016	-	
25	*Aedes japonicus*	Male	8	Otaru aquarium	20th Jul 2016	+	nonspecific reaction
26	*Aedes japonicus*	Male	20	Otaru aquarium	20th Jul 2016	-	
27	*Aedes japonicus*	Male	20	Otaru aquarium	20th Jul 2016	-	
28	*Aedes japonicus*	Male	20	Otaru aquarium	20th Jul 2016	-	
29	*Aedes japonicus*	Male	7	Otaru aquarium	25th Aug 2016	-	
30	*Aedes japonicus*	Male	3	Otaru aquarium	12th Sep 2016	-	
31	*Aedes japonicus*	Female	1	Otaru aquarium	6th Jul 2016	-	
32	*Aedes japonicus*	Female	7	Otaru aquarium	20th Jul 2016	-	
33	*Aedes japonicus*	Female	20	Otaru aquarium	11th Aug 2016	-	
34	*Aedes japonicus*	Female	20	Otaru aquarium	11th Aug 2016	-	
35	*Aedes japonicus*	Female	20	Otaru aquarium	11th Aug 2016	-	
36	*Aedes japonicus*	Female	1	Otaru aquarium	11th Aug 2016	-	
37	*Aedes japonicus*	Female	15	Otaru aquarium	12th Sep 2016	-	
38	*Aedes japonicus*	Female	20	Otaru aquarium	12th Sep 2016	-	
39	*Aedes japonicus*	Female	20	Otaru aquarium	27th Sep 2016	-	
40	*Aedes japonicus*	Female	20	Otaru aquarium	27th Sep 2016	-	
41	*Aedes japonicus*	Male	1	Rakuno Gakuen Univ.	15th Jul 2020	-	

42	*Aedes japonicus*	Female	10	Rakuno Gakuen Univ.	24th Jun 2020	-
43	*Aedes japonicus*	Female	8	Rakuno Gakuen Univ.	24th Jun 2020	-
44	*Aedes japonicus*	Female	10	Rakuno Gakuen Univ.	14th Jul 2020	-
45	*Aedes japonicus*	Female	1	Rakuno Gakuen Univ.	14th Jul 2020	-
46	*Aedes japonicus*	Female	8	Rakuno Gakuen Univ.	15th Jul 2020	-
47	*Aedes japonicus*	Female	1	Rakuno Gakuen Univ.	15th Jul 2020	-
48	*Aedes japonicus*	Female	10	Rakuno Gakuen Univ.	20th Aug 2020	-
49	*Aedes japonicus*	Female	10	Rakuno Gakuen Univ.	20th Aug 2020	-
50	*Aedes japonicus*	Female	8	Rakuno Gakuen Univ.	20th Aug 2020	-
51	*Aedes japonicus*	Female	10	Rakuno Gakuen Univ.	15th Jul 2020	-
52	*Aedes japonicus*	Female	10	Rakuno Gakuen Univ.	15th Jul 2020	-
53	*Aedes japonicus*	Female	9	Rakuno Gakuen Univ.	15th Jul 2020	-
54	*Aedes japonicus*	Female	1	Rakuno Gakuen Univ.	15th Jul 2020	-
55	*Aedes japonicus*	Male	4	Nopporo Forest Park	20th Aug 2020	-
56	*Aedes japonicus*	Female	4	Nopporo Forest Park	20th Aug 2020	-
57	*Aedes japonicus*	Female	6	Nopporo Forest Park	8th Sep 2020	-
58	*Aedes japonicus*	Female	3	Nopporo Forest Park	20th Aug 2020	-
59	*Aedes hokkaidensis*	Female	1	Rakuno Gakuen Univ.	24th Jun 2020	-
60	*Aedes hokkaidensis*	Female	1	Rakuno Gakuen Univ.	24th Jun 2020	-
61	*Aedes hokkaidensis*	Female	1	Rakuno Gakuen Univ.	24th Jun 2020	-
62	*Aedes hokkaidensis*	Female	2	Rakuno Gakuen Univ.	14th Jul 2020	-
63	*Aedes galloisi*	Male	3	Nopporo Forest Park	20th Aug 2020	-

64	*Aedes galloisi*	Male	3	Nopporo Forest Park	20th Aug 2020	-	
65	*Aedes galloisi*	Male	4	Nopporo Forest Park	20th Aug 2020	-	
66	*Aedes galloisi*	Male	1	Nopporo Forest Park	8th Sep 2020	-	
67	*Aedes galloisi*	Female	3	Nopporo Forest Park	20th Aug 2020	-	
68	*Aedes galloisi*	Female	2	Nopporo Forest Park	20th Aug 2020	-	
69	*Aedes galloisi*	Female	2	Nopporo Forest Park	20th Aug 2020	-	
70	*Aedes galloisi*	Female	1	Nopporo Forest Park	20th Aug 2020	-	
71	*Aedes galloisi*	Female	2	Nopporo Forest Park	8th Sep 2020	-	
72	*Aedes galloisi*	Female	3	Nopporo Forest Park	8th Sep 2020	-	
73	*Aedes galloisi*	Female	1	Nopporo Forest Park	8th Sep 2020	-	
74	*Aedes galloisi*	Female	2	Nopporo Forest Park	20th Aug 2020	+	nonspecific reaction*
75	*Aedes albopictus*	Female	2	laboratory-colony	30th Sep 2020	-	
76	*Aedes albopictus*	Female	2	laboratory-colony	30th Sep 2020	-	
77	*Aedes albopictus*	Female	2	laboratory-colony	30th Sep 2020	-	
78	*Aedes albopictus*	Female	2	laboratory-colony	30th Sep 2020	-	
79	*Aedes albopictus*	Female	2	laboratory-colony	30th Sep 2020	-	
80	*Aedes aegypti*	Female	2	laboratory-colony	30th Sep 2020	-	
81	*Aedes aegypti*	Female	2	laboratory-colony	30th Sep 2020	-	
82	*Aedes aegypti*	Female	2	laboratory-colony	30th Sep 2020	-	
83	*Aedes aegypti*	Female	2	laboratory-colony	30th Sep 2020	-	
84	*Aedes aegypti*	Female	2	laboratory-colony	30th Sep 2020	-	
85	*Culex pipiens* group	Male	1	Rakuno Gakuen Univ.	Sep to Oct 2020	-	

86	*Culex pipiens* group	Male	1	Rakuno Gakuen Univ.	Sep to Oct 2020	-	
87	*Culex pipiens* group	Male	1	Rakuno Gakuen Univ.	Sep to Oct 2020	-	
88	*Culex pipiens* group	Female	10	Rakuno Gakuen Univ.	Sep to Oct 2020	+	nonspecific reaction
89	*Culex pipiens* group	Female	10	Rakuno Gakuen Univ.	Sep to Oct 2020	+	nonspecific reaction
90	*Culex pipiens* group	Female	10	Rakuno Gakuen Univ.	Sep to Oct 2020	+	nonspecific reaction
91	*Culex pipiens* group	Female	10	Rakuno Gakuen Univ.	Sep to Oct 2020	+	nonspecific reaction
92	*Culex pipiens* group	Female	10	Rakuno Gakuen Univ.	Sep to Oct 2020	+	nonspecific reaction
93	*Culex pipiens* group	Female	10	Rakuno Gakuen Univ.	Sep to Oct 2020	+	nonspecific reaction
94	*Culex pipiens* group	Female	4	Rakuno Gakuen Univ.	Sep to Oct 2020	-	
95	*Culex orientalis*	Female	3	Rakuno Gakuen Univ.	Sep to Oct 2020	-	
96	*Culex orientalis*	Female	3	Rakuno Gakuen Univ.	Sep to Oct 2020	-	
97	*Culex orientalis*	Female	3	Rakuno Gakuen Univ.	Sep to Oct 2020	+	nonspecific reaction*
98	*Culex orientalis*	Female	3	Rakuno Gakuen Univ.	Sep to Oct 2020	-	
99	*Culex orientalis*	Female	3	Rakuno Gakuen Univ.	Sep to Oct 2020	-	
100	*Culex orientalis*	Female	3	Rakuno Gakuen Univ.	Sep to Oct 2020	-	
101	*Culex orientalis*	Female	3	Rakuno Gakuen Univ.	Sep to Oct 2020	+	nonspecific reaction*
102	*Culex orientalis*	Female	2	Rakuno Gakuen Univ.	Sep to Oct 2020	+	nonspecific reaction*
103	*Anopheles sineroides*	Female	1	Rakuno Gakuen Univ.	Sep to Oct 2020	-	

*Possibly a reaction against mosquito DNA.
